# Incidence and Characteristics of Bacteremia among Children in Rural Ghana

**DOI:** 10.1371/journal.pone.0044063

**Published:** 2012-09-10

**Authors:** Maja Verena Nielsen, Nimako Sarpong, Ralf Krumkamp, Denise Dekker, Wibke Loag, Solomon Amemasor, Alex Agyekum, Florian Marks, Frank Huenger, Anne Caroline Krefis, Ralf Matthias Hagen, Yaw Adu-Sarkodie, Jürgen May, Norbert Georg Schwarz

**Affiliations:** 1 Infectious Disease Epidemiology, Bernhard Nocht Institute for Tropical Medicine, Hamburg, Germany; 2 Kumasi Centre for Collaborative Research in Tropical Medicine, Kumasi, Ghana; 3 International Vaccine Institute, Seoul, South Korea; 4 Kwame Nkrumah University of Science and Technology, School of Medical Sciences, Kumasi, Ghana; 5 Institute for Transfusion Medicine, Laboratory Medicine and Medical Microbiology, Dortmund, Germany; Oxford University, Viet Nam

## Abstract

The objective of the study was to describe systemic bacterial infections occurring in acutely ill and hospitalized children in a rural region in Ghana, regarding frequency, incidence, antimicrobial susceptibility patterns and associations with anthropometrical data.

Blood cultures were performed in all children below the age of five years, who were admitted to Agogo Presbyterian Hospital (APH), Asante Region, Ghana, between September 2007 and July 2009. Medical history and anthropometrical data were assessed using a standardized questionnaire at admission. Incidences were calculated after considering the coverage population adjusted for village-dependent health-seeking behavior.

Among 1,196 hospitalized children, 19.9% (n = 238) were blood culture positive. The four most frequent isolated pathogens were nontyphoidal salmonellae (NTS) (53.3%; n = 129), *Staphylococcus aureus* (13.2%; n = 32), *Streptococcus pneumoniae* (9.1%; n = 22) and *Salmonella* ser. Typhi (7.0%; n = 17). Yearly cumulative incidence of bacteremia was 46.6 cases/1,000 (CI 40.9–52.2). Yearly cumulative incidences per 1,000 of the four most frequent isolates were 25.2 (CI 21.1–29.4) for NTS, 6.3 (CI 4.1–8.4) for *S. aureus*, 4.3 (CI 2.5–6.1) for *S. pneumoniae* and 3.3 (CI 1.8–4.9) for *Salmonella* ser. Typhi. Wasting was positively associated with bacteremia and systemic NTS bloodstream infection. Children older than three months had more often NTS bacteremia than younger children. Ninety-eight percent of NTS and 100% of *Salmonella* ser. Typhi isolates were susceptible to ciprofloxacin, whereas both tested 100% susceptible to ceftriaxone. Seventy-seven percent of NTS and 65% of *Salmonella* ser. Typhi isolates were multi-drug resistant (MDR). Systemic bacterial infections in nearly 20% of hospitalized children underline the need for microbiological diagnostics, to guide targeted antimicrobial treatment and prevention of bacteremia. If microbiological diagnostics are lacking, calculated antimicrobial treatment of severely ill children in malaria-endemic areas should be considered.

## Introduction

High infant morbidity and mortality is still one of the major health issues in sub-Saharan Africa with 4.6 million children dying before the age of five years [1]. According to WHO statistics of 2008, malaria accounts for 18% of deaths among children below five years in Ghana, closely followed by pneumonia (13%), diarrhea (12%) and pre-maturity at birth (12%). Neonatal sepsis is causing 9% of fatal cases. Apart from a few well-equipped hospitals, health facilities lack microbiological diagnostic capacities necessary to diagnose bacteremia and to isolate bacterial pathogens in order to allow targeted treatment [2]. While public interest tends to focus on malaria, tuberculosis and HIV, the morbidity and mortality burden of systemic bloodstream infections are still insufficiently investigated. Recently published data from Tanzania reveals clinical overestimation of malaria, whereas invasive bacterial disease was underestimated [3]. Clinical differentiation between severe malaria and invasive bacterial infection is difficult because of the overlap in disease symptoms [4]. A further factor complicating the diagnosis and therapy is self-treatment with antimicrobial drugs prior to professional health care, which may impede the diagnosis and increases the risk of emergence and spread of antibiotic resistance [5].

Appropriate diagnosis and treatment require better knowledge of the spectrum of infective agents in malaria-endemic countries as well as the characteristics and disease symptoms associated with the infections. Accordingly, the aim of the study was to provide information on infection incidences, on the spectrum of antibiotic resistances, and on clinical characteristics of bacteremic children.

## Methods

This hospital-based study was carried out at Agogo Presbyterian Hospital (APH) in a rural area of the Ashanti Region in Ghana. The catchment area encompasses approximately 149,500 people [6] with more than 28,000 inhabitants living in Agogo town [7]. The territory was originally covered by tropical rainforest that gave yield to secondary forest, bush land and crop acreages as a result of increased logging and farming activity in the last decades. Two rainy seasons, from May to July and from September to October, characterize the climate with an air humidity up to 80%, ambient temperatures between 28–36°C during daytime and an average temperature of 24°C at night.

Fifteen percent of the population is aged below five years according to census data of 2004. WHO data from 2008 demonstrates a high mortality of 76 per 1,000 children below five years of age. The Ashanti Region is an area holoendemic for malaria with reported transmission rates of >100 cases per 1,000 inhabitants in 2008 [8]. A National Health Insurance Scheme (NHIS) was officially launched in March 2004, covering 38% of the district population in 2009 [9]. The HIV prevalence was 1.8% for the adult Ghanaian population in 2009 [10]. In 2001, *Haemophilus influenzae* type B conjugate vaccine was introduced in the immunization schedule of children [11].

### Data collection and definition of variables

A survey of consecutive visits of patients presenting at the outpatient department of APH was conducted between September 2007 and July 2009. Ethical approval for the study was obtained from the Committee on Human Research, Publications, and Ethics, School of Medical Science, Kwame Nkrumah University of Science and Technology (KNUST), Kumasi, Ghana. Aims and principles of the study were explained in detail to participants and informed consent was sought by signature or thumb print by the caregiver. Included were all patients up to the age of five years including neonates, who were admitted to the children's ward, provided that parents or legal guardians accepted the study conditions and signed or thumb printed the written informed consent document. Children with dermatological or surgical conditions obviously not caused by a systemic infection were excluded.

Data collection was embedded into clinical routine. Personal and anthropometrical data was collected using a four-paged admission sheet, that was filled in by doctors or study nurses and subsequently double entered by two independent data entry clerks using a 4^th^ Dimension Database 2004.4 © 4D SA, 1985–2006. (Clichy-la-Garenne, France).

Between one and three milliliter venous blood was taken from every child for blood cultures, inoculated into blood culture bottles (Becton Dickinson (BD) BACTEC™ PEDS PLUS™/F) and incubated using an automated BACTEC™ 9050 Blood Culture System (BD, Franklin Lakes, NJ USA) for five days or until positive. For bacterial identification, all positive blood cultures were examined directly by Gram stain microscopy and subcultured on standard media plates. Identification of the organisms was obtained by biochemical and serological tests. Every child, who grew at least one pathogenic bacterial organism, was considered to be bacteremic. Isolates of non-pathogenic microorganisms or skin flora (e.g. *coagulase-negative Staphylococci*, *Propionibacterium* spp., *Corynebacterium* spp. and *Bacillus* spp. other than *Bacillus anthracis*) were considered to be contaminants. Contaminants were included in the calculation of frequencies, yet excluded from further bacteremia analyses. Susceptibility to penicillin, amoxicillin/ampicillin, amoxiclav (amoxicillin & clavulanic acid), flucloxacillin, cefuroxime, ceftriaxone, erythromycin/azithromycin, cotrimoxazole, ciprofloxacin, gentamicin, tetracycline and chloramphenicol was tested using the Kirby-Bauer disc diffusion method.

Multi-drug resistance of *Salmonella enterica* was defined as simultaneous resistance to amoxicillin, cotrimoxazole and chloramphenicol. *S. enterica* were screened for resistance to fluoroquinolones (FQ) by nalidixic acid disc diffusion following the Clinical and Laboratory Standards Institute (CLSI) guidelines of 2011. Nalidixic acid resistant strains were further tested by ciprofloxacin E test.

To assess the nutritional status of the children, we calculated the Z-scores of the anthropometric indices weight-for-age (underweight), weight-for-height (wasting) and height/length-for-age (stunting) using 2006 WHO child growth standards. Cut-off points were Z-scores of ±2 as suggested by WHO in 1997.

After physical examination, every child obtained standardized malaria diagnostics, including a thick and a thin smear from capillary blood samples. In case a child was parasitemic and presented with typical clinical signs of malaria, it received national first-line treatment for uncomplicated *Plasmodium falciparum* malaria.

### Descriptive analysis

Categorical variables were described using frequencies along with percentages. Continuous variables were displayed using the mean, the standard deviation (SD) and the maximum and minimum values. For non-normally distributed variables the median along with the range were reported.

### Calculation of yearly incidence

The recorded number of bacteremic infections (C_i_) during the observation period was divided by the number of children below five years living in the hospital catchment area (Pop_<5_), derived from census data of 2004. Absolute numbers of each village were corrected for the proportion of people, who reported that they would access the study hospital in case of illness (*p*). These figures were retrieved from a hospital utilization survey, conducted throughout the hospital catchment area in 2007 (results not shown). The comparison of official hospital admission records with our case records showed that about 50% of hospitalized patients potentially matching the inclusion criteria were recruited. Therefore, the number of identified cases was weighted by the factor 0.5 (*F_miss_*). To retrieve the annual incidence, the figures were divided by the observation period of 23 months given in months (*m*) and multiplied by 12. Thus, the annual cumulative incidences of bacterial infections were calculated via the equation 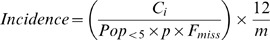
(1)


where


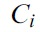
 =  Recorded number of bacteremic infections


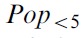
 =  Number of children below five years living in the hospital catchment area


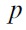
 =  Proportion of people reporting they would access the study hospital in case of illness


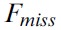
 =  Weighting by the factor 0.5 to adjust for missing recruitments


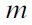
 =  Obsezrvation period of 23 months. 

Since the incidence is based on the proportion of infected children from the total number of children who would seek health care at APH, the corresponding confidence intervals were calculated using the standard error for proportions.

### Analytical procedure

In the analytical part of the study the association of bacteremia with sex, age, parasitemia and anthropometric data (i.e. underweight, wasting, stunting) was estimated on the basis of a case-control design. Controls were all children with a negative blood culture. The crude effects were described using Odds Ratios (ORs) and the corresponding 95% confidence intervals (CIs). A logistic regression model was calculated to show the adjusted effects of the different variables. Model variable selection was done backwards, using likelihood ratio tests and content wise, considered proven association. Only observations without missing data were considered for the regression models in order to be able to conduct the diagnostic tests. The data analysis was carried out using STATA 10 software (College Station, TX: StataCorp LP).

## Results

In total, 1,351 children below five years, admitted to the Children's ward between September 2007 and July 2009, were included in the study. One hundred fifty-five were excluded from the analysis because of missing data on blood cultures, leaving 1,196 hospitalized children. In 116 (9.7%) children, blood culture isolates were considered as contaminants. Pathogens were isolated in 238 (19.9%) cases. In four of these children, two pathogens were detected simultaneously in one culture, leading to a total number of 242 bacterial isolates.

Overall, nontyphoidal salmonellae (NTS) were the predominant bacterial pathogens with 129 (53.3%) isolates in total. *Staphylococcus aureus* constituted the second frequent isolate (13.2%, n = 32), followed by *Streptococcus pneumoniae* (9.1%, n = 22). Seventeen infections (7.0%) were caused by *Salmonella* ser. Typhi. The full list of pathogens is given in Table 1.

**Table 1:** Frequency and estimated incidence of bacteremia in the hospital catchment area in 1,196 children below five years of age.

Pathogen	Total frequency	Incidence^a^	Age (months) stratified frequencies (%)					
			<1	1–11	12–23	24–35	36–47	48–60
Bacteremic children^b^	238 (19.9)	46.6 (40.9–52.2)	23 (19.3)	76 (18.9)	72 (22.9)	31 (16.9)	24 (23.8)	12 (15.6)
Pathogen isolates (n = 242)^c^								
Nontyphoidal salmonellae	129 (53.3)	25.2 (21.1–29.4)	2 (8.7)	46 (59.7)	45 (62.5)	18 (56.3)	15 (57.7)	3 (25.0)
*Staphylococcus aureus*	32 (13.2)	6.3 (4.1–8.4)	6 (26.1)	9 (11.7)	10 (13.9)	2 (6.3)	3 (11.5)	2 (16.7)
*Streptococcus pneumoniae*	22 (9.1)	4.3 (2.5–6.1)		10 (13.0)	5 (6.9)	3 (9.4)	3 (11.5)	1 (8.3)
*Salmonella* ser. Typhi	17 (7.0)	3.3 (1.8–4.9)			4 (5.6)	4 (12.5)	4 (15.4)	5 (41.7)
*Klebsiella* spp.	10 (4.1)		6 (26.1)	2 (2.6)	2 (2.8)			
*Streptococcus* spp.	9 (3.7)		3 (13.0)	3 (3.9)	1 (1.4)	1 (3.1)	1 (3.8)	
*Escherichia coli*	8 (3.3)		3 (13.0)	1 (1.3)	1 (1.4)	2 (6.3)		1 (8.3)
*Acinetobacter* spp.	4 (1.6)		1 (4.3)	1 (1.3)	1 (1.4)	1 (3.1)		
*Pseudomonas* spp.	2 (0.8)			1 (1.3)		1 (3.1)		
*Haemophilus* spp.	1 (0.4)			1 (1.3)				
*Aeromonas hydrophila*	1 (0.4)		1 (4.3)					
*Morganella morganii*	1 (0.4)				1 (1.4)			
*Enterococcus* spp.	1 (0.4)			1 (1.3)				
*Pantoea* spp.	1 (0.4)		1 (4.3)					
Other bacteria	4 (1.6)			2 (2.6)	2 (2.8)			

a Yearly cumulative incidence per 1,000.

b Isolates considered as contaminants: n = 116 (9.7%).

c Double infections in 4 children: NTS/*S. pneumoniae*, NTS/*S. aureus*, *Salmonella* ser. Typhi/*Streptococcus* spp., *S. aureus*/*S. pneumoniae*.

One hundred (19.2%) of 521 children aged up to 12 months were bacteremic. Below the age of one month, 23 of 119 children (19.3%) had positive blood cultures. The most frequent isolates found in this subgroup were *S. aureus* (26.1%, n = 6), *Klebsiella* spp. (26.1%, n = 6), *Streptococcus* spp. (other than *S. pneumoniae*) (13.0%, n = 3) and *Escherichia coli* (13.0%, n = 3).

The calculated reference population of children younger than five years was 5,333, which were approximately 20% of the children under five years who were living in the hospital catchment area. The annual cumulative incidence of bacteremia was 46.6 cases/1,000 children (CI 40.9–52.2). The annual cumulative incidence per 1,000 children of the four most frequent isolates was 25.2 (CI 21.1–29.4) for NTS, 6.3 (CI 4.1–8.4) for *S. aureus*, 4.3 (CI 2.5–6.1) for *S. pneumoniae*, and 3.3 (CI 1.8–4.9) for *Salmonella* ser. Typhi (Table 1).

### Outcome of illness at time of discharge

The outcome of bacteremia at the time of discharge was known in 121 (50.6%) children. However, other underlying illnesses that may have contributed to the clinical expression of outcome could not completely be excluded. The case-fatality rate of children with bacteremia was 9.1% (n = 11) and 13.2% (n = 16) were reported to suffer from further on-going disabilities after discharge. Only 4.8% (n = 27) of non-bacteremic cases were fatal and 12.0% (n = 68) presented with prolonged disabilities. Isolates from fatal cases were four NTS, three *S. pneumoniae*, two *Klebsiella* spp., one *Salmonella* ser. Typhi and one *Acinetobacter* spp. The mean age of fatal cases with bacteremia was 15.4 months (SD ±18.0), which was similar to the mean age of children without bacteremia (13.1 months, SD ±16.0).

### Susceptibility to antimicrobials

According to interviews with the guardians, 679 (65.6%) children received paracetamol, 237 (23.9%) antimalarials, and 223 (25.9%) antihelminths before seeking help at APH. Altogether, 1,044 (95.3%) children received any kind of oral medication prior to hospitalization. Only 68 (9.4%) of the guardians could report specific antimicrobial treatment. Fourty-seven (69.1%) used amoxicillin, five (7.4%) cefuroxime/ceftriaxone, four (5.9%) flucloxacillin, four (5.9%) cloxacillin, two (2.9%) erythromycin and 6 (8.8%) metronidazole, cotrimoxazole, chloramphenicol or ampicillin/gentamicin.

Susceptibility of the four most frequent isolated organisms to antimicrobial drugs is listed in Table 2. The best coverage for all four organisms was achieved by using ceftriaxone, to which all NTS, *S. pneumoniae* and *Salmonella* ser. Typhi isolates were susceptible. Similar coverage was seen for ciprofloxacin with a 100%-susceptibility in *Salmonella* ser. Typhi and NTS. However, two NTS isolates tested resistant to nalidixic acid by disc diffusion and showed intermediate level of resistance by ciprofloxacin E-test. Only 12 (57.1%) and 21 (67.7%) of the *S. pneumoniae* and *S. aureus* isolates were susceptible to ciprofloxacin, respectively. MDR (i.e. resistance against amoxicillin, chloramphenicol and cotrimoxazole) was observed in 75 (77.0%) of NTS and 11 (65%) of *Salmonella* ser. Typhi isolates.

**Table 2:** Frequency and proportion of susceptibility of the four most frequent isolated pathogens to antibiotics and MDR^a^.

	NTS		*S. aureus*		*S. pneumoniae*		*Salmonella* ser. Typhi	
	(n = 129)		(n = 32)		(n = 22)		(n = 17)	
	Susceptible	%	Susceptible	%	Susceptible	%	Susceptible	%
Penicillin	-	-	15/31	48	16/21^a^	76	-	-
Amoxicillin/Ampicillin	18/123	15	-	-	17/22^a^	77	6/17	35
Amoxicillin-Clavulanate	28/114	25	-	-	-	-	10/14	71
Flucloxacillin	-	-	25/30	83	-	-	-	-
Cefuroxime	-	-	-	-	6/6	100	-	-
Ceftriaxone	108/108	100	-	-	17/17	100	17/17	100
Erythromycin/Azithromycin	-	-	21/31	68	20/20	100	-	-
Cotrimoxazole	22/98	22	13/24	54	1/20	5	5/17	29
Ciprofloxacin	127/127	100	21/31	68	12/21	57	17/17	100
Nalidixic Acid	125/127	98^b^	-	-	-	-	17/17	100
Gentamicin	-	-	22/31	71	5/20	25	-	-
Tetracycline	83/91	91	8/21	35	5/19	26	7/16	44
Chloramphenicol	22/127	17	2/31	6	15/20	75	5/17	29
MDR^c^	75/98	77	-	-	-	-	11/17	65

a One strain showed discrepant susceptibility results to penicillin and to amoxicillin/ampicillin and was excluded because the strain was not available for further analyses.

b Two strains tested resistant to nalidixic acid by disc diffusion and showed intermediate level of resistance to ciprofloxacin by the E test method.

c MDR (multi drug resistance; resistance to amoxicillin, cotrimoxazole and chloramphenicol) among nontyphoidal salmonellae (NTS) and *Salmonella* ser. Typh.

### Association of anthropometrical parameters with bacteremia and systemic NTS infection

The proportion of female (53.8%) and male (46.2%) children in the study population was similar. The age pattern was left skewed (median 14 months). Five hundred twenty-one (43.6%) children were younger than one year, and 119 (9.9%) were younger than one month. The frequency of children being underweight, wasted or stunted was 267 (26.3%), 172 (24.8%) and 146 (20.9%), respectively. Two hundred and four (29.5%) children with complete malaria diagnostics were diagnosed with *Plasmodium falciparum* parasitemia.

In a case-control study, the association of six parameters (sex, age, parasitemia, underweight, wasting, and stunting) with bacteremia and, specifically, NTS bacteremia were analyzed (Table 3). Other isolates were not considered due to small case numbers. In the crude analysis, no associations between bacteremia and sex or age were observed. Bacterial infection rates in females and males were similar (OR = 1.0, CI = 0.8–1.4). The median age for cases (bacteremia) and controls (no bacteremia) was 13 vs. 14 months, respectively. The total frequency of hospitalized children with bacteremia decreased with age. Stratified for age, the proportion of positive blood cultures undulated around 20% (range = 15.6%–23.8%), whereas the proportion of contaminated samples decreased with age.

**Table 3:** Crude associations of anthropometrical parameters and parasitemia with bacteremia in children below five years of age in Ghana^a^.

		Bacteremia		
		No	Yes (%)	OR (CI)^b^
Sex (n = 1,196)	Female	517	126 (19.6)	1
	Male	441	112 (20.3)	1.0 (0.8–1.4)
Age (n = 1,196)	<3 month	179	39 (17.9)	1
	3–5 months	69	17 (19.8)	1.1 (0.6–2.1)
	6–11 months	174	43 (19.8)	1.1 (0.7–1.8)
	12–23 months	242	72 (22.9)	1.4 (0.9–2.1)
	24–60 months	294	67 (18.6)	1.0 (0.7–1.6)
Parasitemia (n = 691)	No	388	99 (20.3)	1
	Yes	174	30 (14.7)	0.7 (0.4–1.1)
Underweight (n = 1,015)	No	625	123 (16.4)	1
	Yes	197	70 (26.2)	1.8 (1.3–2.5)
Wasting (n = 695)	No	440	83 (15.9)	1
	Yes	127	45 (26.2)	1.9 (1.2–2.8)
Stunting (n = 700)	No	457	97 (17.5)	1
	Yes	115	31 (21.2)	1.3 (0.8–2.0)

a Cases with missing data for respective parameters were excluded from the analysis.

b OR (CI), Odds Ratio (95% confidence interval).

The frequency of NTS infections differed between the age groups. The highest risk of NTS infection was observed in the age groups six to eleven (OR = 4.2, CI = 1.9–9.4) and 12 to 23 months (OR = 4.4, CI = 2.0–9.5) in which 13.8% (n = 30) and 14.3% (n = 45) of the children had infections with NTS. In the age group zero to five months, only 5.9% (n = 18) of the blood cultures were positive for NTS. Again, there was no association between NTS infection and sex (OR 1.0, CI 0.7–1.5).

Parameters for underweight and wasting, indicators for acute malnutrition, were associated with bacteremia (OR = 1.8, CI = 1.3–2.5 and OR = 1.9, CI = 1.2–2.8, respectively). Similar patterns were shown for the NTS group (OR = 1.6, CI = 1.1–2.5 and OR = 2.0, CI = 1.2–3.4, respectively). Stunting, an anthropometrical parameter to describe chronic malnutrition, did not show any association. No association among parasitemia and bacteremia nor parasitemia and NTS was observed (Table 3).

Logistic regression models were generated to describe the adjusted associations with bacteremia and NTS. Finally, 695 (58.1%) of observations could be used for logistic regression. In the crude analysis, underweight and wasting were associated with the outcome variable. Yet both conditions were constructed via Z-scores, which are based on children's weight, leading to autocorrelation (OR = 24.6, CI = 15.5–39.3). Since wasting is most important for clinical diagnosis, underweight was not considered for the regression models.

Older age categories had higher odds for infection compared to the youngest age group (<3 months), and children being wasted were more likely to carry bacterial infections (Table 4). Likewise for NTS, the adjustment through regression did not change the results of the crude analysis substantially. High odds ratios for the above-mentioned age categories were shown and wasting remained associated within the regression model.

**Table 4:** Adjusted associations of anthropometrical parameters and parasitemia with bacteremia in children below five years of age in Ghana.

		Bacteremia
		OR (CI)^a^
Age	<3 months	1
	3–5 months	1.8 (0.7–4.3)
	6–11 months	1.5 (0.7–3.1)
	12–23 months	1.9 (0.9–3.7)
	24–60 months	1.8 (0.9–3.6)
Wasting	No	1
	Yes	2.0 (1.3–3.0)

a OR (CI), Odds Ratio (95% confidence interval).

## Discussion

Bacterial bloodstream infections were detected in one-fifth of hospitalized children leading to an estimated yearly incidence of 46.6/1,000. This result underlines the relevance of bacterial infections as differential diagnosis of severe febrile illness in malaria endemic areas. Our estimated incidences were five to ten times higher than incidence estimates from other parts of sub-Saharan Africa. A study from Kenya on community-acquired bacteremia in children used the total age population of the catchment area to obtain what the authors described as “minimal incidence estimates” [12]. In contrast, we acquired data on health-seeking behavior from a community survey, which was performed in the study area, and accordingly adapted the denominators for our incidence estimates on the village level only including the population proportion of each village that reported to seek health care at our study hospital. The prevalences of bacteremic children in Kenya [13], Mozambique [14] and Tanzania [3], [15], who were admitted to the hospital with either febrile illness or signs of severe malaria, were reported in the range between 5.8% and 9.4%, which was two to four times lower when compared to our findings. Stratified for plasmodium parasitemia, the prevalence of bacteremia in non-parasitemic children increased to 18% in Kenya and 16.7% in Tanzania compared to prevalences in parasitemic children, ranging between 4.6% and 6.2%. Considering the malaria prevalence on admission in these regions, being with more than 60% twice as high than at APH (13.8%–30.6% [16]), the reason for higher proportions of children with bacteremia at APH might be the lower prevalence of malaria as important differential diagnosis in these children with severe illness.

High total yearly incidences of bacteremia and especially invasive NTS infection demonstrate the impact of septicemia on children's morbidity in this area. This confirms results from Kumasi (2004) showing similarly high proportions of 20.3% bacteremia in children with signs of severe malaria [4]. Reasons, in particular for the high NTS infection rates, yet, remain unclear and deserve further investigation.

In our setting, NTS accounted for more than half of invasive bacterial infections (53.1%) in children below the age of five years, and NTS infection was positively associated with clinical malnutrition (i.e. wasting; OR = 1.9, CI = 1.2–3.4) and age above three months when compared to younger children. Similarly, high infection rates for NTS were found in Tanzania [15] reporting 52% NTS in malaria slide positive children. Bacteremia in general did not show this distinct age trend. In the NTS group, highest levels of infection were reached in infants after time of breast-feeding, possibly marking the onset of intake of contaminated weaning food and water [17], [18].

Poor socio-economic status and chronic malnutrition could impair the immune response and predispose to invasive infection with NTS. In the latter case, however, one would expect an association with stunting (OR = 1.2, CI = 0.6–2.2), a parameter for chronic malnutrition.

**Figure 1:**
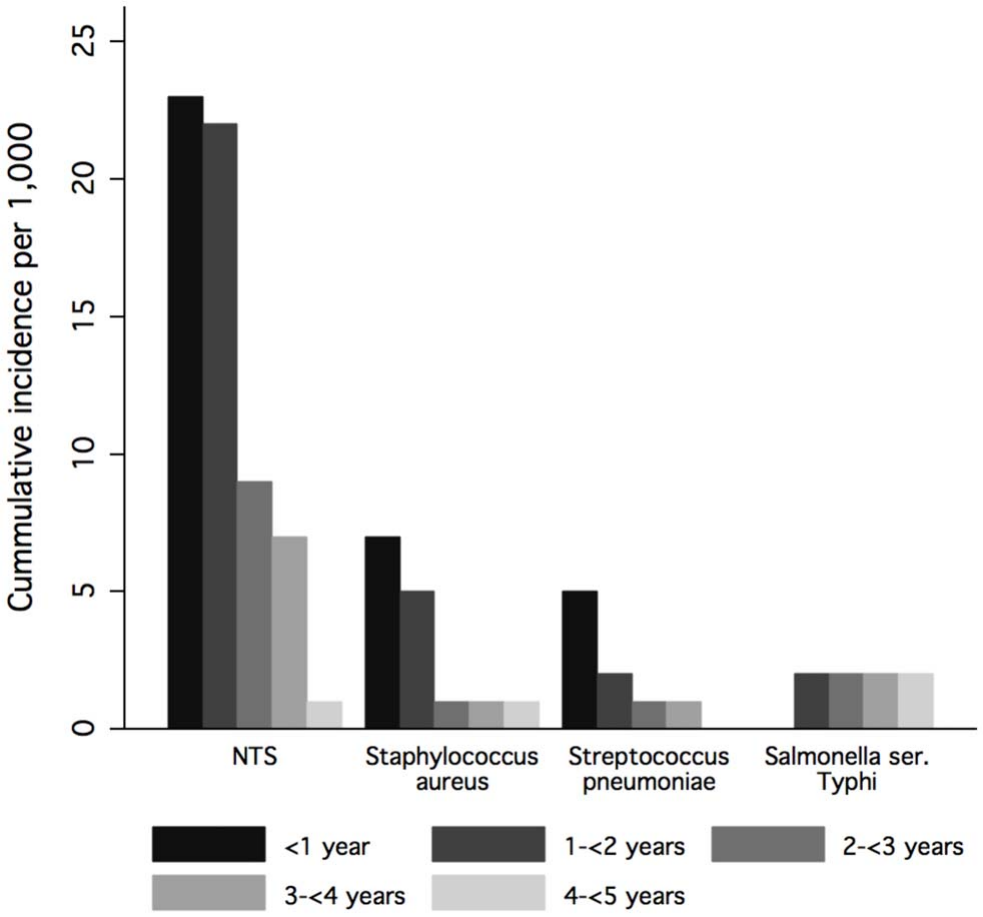
Incidence of isolates (nontyphoidal salmonella (NTS), *Staphylococcus aureus*, *Streptococcus pneumoniae*, *Salmonella* ser. Typhi) stratified by age.

*S. aureus* was the second most frequent isolate in our study. More than three quarters (78.1%) of all *S. aureus* isolates were found in children below two years of age, nearly half of them in infants under one year of age (46.9%) (Figure 1). This age distribution is typical for *S. aureus* in sub-Saharan Africa [19], [20]. The fact that this pathogen often affects young children and neonates makes it particularly dangerous, especially if isolates are methicillin resistant. Antibiotic susceptibility testing revealed that already 17% of our isolates showed resistance to methicillin. None of the antibiotics tested were effective against all *S. aureus* isolates.

Data from Kenya [12] and The Gambia [21] found a predominance of *S. pneumoniae* in children. Indeed, *S. pneumoniae* was estimated to be the leading bacterial cause of death in young children worldwide in the year 2000 [22] and was described in a recent meta-analysis as the most common isolate in children of community-acquired bloodstream infections in African countries [23]. In our study group, *S. pneumoniae* accounted for 9.1% of the identified pathogens. Previously, only one (2%) *S. pneumoniae* case was detected in another study carried out in Kumasi in 2004 [4]. The authors explained the low isolation rate with laboratory difficulties and previous antimicrobial self-medication of the patients. Other studies from Kumasi [24] and Accra [25] underlined the importance of invasive pediatric disease with *S. pneumoniae* in Ghana and advocated the importance of adjusted vaccination.

*Salmonella* ser. Typhi accounted for 7.1% of all isolated pathogens and thus fell under the four most important bacterial pathogens in children with systemic bacterial infection. This outcome is nearly congruent with earlier published results [26]. Typhoid fever vaccinations are available, however, they are not routinely used in sub-Saharan Africa but under consideration and regarded to be potentially cost-effective [27].

The only *Haemophilus influenzae* isolate in our study was found in a two months old boy that had only received BCG immunization until the onset of disease. The low incidence of *H. influenzae*, known to be the cause of pneumonia, meningitis and epiglottitis, especially in children below the age of three years [28], might reflect the success of vaccination programs.

Antibiotic resistance is an increasing problem in Ghana [29], [30] and in other African countries [31] and often causes fatal outcomes [32]. This study has shown that MDR is very high in respective groups of bacteria but all NTS strains were still sensitive to ceftriaxone. Two NTS isolates (1.6%) were resistant to nalidixic acid and showed intermediate levels of resistance to ciprofloxacin. This might be first indication that resistance to FQ could become more important in the future in Ghana. The methods used for the detection of drug resistances followed the CLSI guidelines valid in 2011, and nalidixic acid was used as the screening method for the detection of FQ resistance. To note, in the new guidelines from 2012, it is recommended to use the ciprofloxacin E-test, as FQ resistance may be missed by exclusive nalidixic acid testing.

Due to relatively low susceptibility of *S. pneumoniae* (57%) to ciprofloxacin, this drug should be used cautiously as calculated antimicrobial therapy in children with clinical evidence of pneumococcal infections. In addition, recent data from Ghana underlines that sepsis with NTS should be considered in children with respiratory symptoms. NTS were the predominant organisms isolated from children with clinical pneumonia and significantly more frequent than *S. pneumoniae* [33]. In this case, ceftriaxone may be an alternative, as it fully covers NTS, *Salmonella* ser. Typhi and *S. pneumoniae*. The exclusive intravenous use of ceftriaxone may be limiting under resource-poor conditions, but otherwise protects from uncontrolled intake and the development of resistance. A recent study carried out in Mozambique in 2010 similarly suggests that quinolones (e.g. ciprofloxacin) and third generation cephalosporins (e.g. ceftriaxone) may be required to manage community-acquired infections [34]. However, from other regions of the world, resistance of NTS to ceftriaxone has already been reported, which might be a consequence of widespread use of ceftriaxone [35] as a first line-drug treatment.

Routine antimicrobial treatment in children with signs of severe malaria has been discussed controversially [36], . Yet a clear association between malaria and bacteremia has not been proven [37]. In opposite to findings from The Gambia, which suggest an association between malaria and NTS by comparing temporal trends [38], NTS infection and *P. falciparum* parasitemia did not reveal any positive association in our setting. However, children caught in the vicious circle of poor socio-economic status, malnutrition and a tendency to develop systemic infection due to weak immune response may have both, a higher risk of severe malaria and of complicating bacterial co-infection.

Blood sampling in infants and young children is particularly difficult, and the amount of blood that can be sampled tends to be lower than required. Small volumes of inoculated blood and the use of antimicrobial treatment previous to blood sampling may compromise blood culture sensitivity and antimicrobial susceptibility testing. Technical difficulties are reflected in the decreasing frequency of contaminants with age. Thus we have to assume that there is a tendency to underestimate the burden of systemic bacterial infections in small children. Furthermore, by considering all children with non-pathogenic cultural isolates as negative, some true cases of sepsis might have been missed. Since the study was embedded into clinical routine, random loss of data on different operational levels occurred. The heterogeneous pattern of patients with missing data makes a significant selection bias unlikely. Reasons were e.g. technical difficulties and admissions at night resulting in missed documentation of clinical outcome and anthropometrical parameters.

## Conclusion

Community-acquired bacteremia is an important differential diagnosis of malaria in Ghanaian children below the age of five and is closely linked to age and nutritional status of the child. If microbiological diagnostics are lacking, preventive antimicrobial treatment of severely ill children in malaria-endemic areas should be considered, particularly in children presenting with clinical signs of malnutrition. The data emphasizes the need for implementation of food and water sanitation besides the establishment of laboratory facilities for diagnostic testing. Moreover, it underlines the necessity of focusing research on child health programs as well as on vaccination against predominant organisms of systemic bacterial infections in African children.
